# Maternal vitamin D status in early pregnancy and its association with gestational diabetes mellitus in Shanghai: a retrospective cohort study

**DOI:** 10.1186/s12884-022-05149-1

**Published:** 2022-11-05

**Authors:** Yan Cheng, Jiayuan Chen, Tingting Li, Jiangnan Pei, Yongfang Fan, Mulan He, Shuangping Liu, Junxiu Liu, Qingying Zhang, Haidong Cheng

**Affiliations:** 1 Obstetrics & Gynecology Hospital of Fudan University, 128 Shenyang Road, Shanghai, 200090 China; 2 Zhongshan hospital, Fudan university (Xiamen branch), Jinhu Road 668, Xiamen, 361015 China

**Keywords:** Vitamin D status, Gestational diabetes mellitus, Serum 25(OH)D, Pregnant women

## Abstract

**Background:**

There is growing interest regarding vitamin D and its potential role in gestational diabetes mellitus (GDM). We aimed to assess maternal vitamin D status in early pregnancy and its relationships with the risk of GDM in a Chinese population in Shanghai.

**Methods:**

The retrospective cohort study included a total of 7816 pregnant women who underwent a 75-g oral glucose tolerance test (OGTT) during 24–28 weeks of gestation. Participants’ demographic information including maternal age, prepregnancy body mass index (BMI), gestational age, parity, season of blood collection, serum 25-hydroxy vitamin D [25(OH)D] data and other blood biomarker data at 6 to 14 weeks of gestation were retrospectivly extracted from the medical records in the hospital information system.

**Results:**

In the cohort, the prevalence of GDM was 8.6% and the prevalence of vitamin D deficiency and insufficiency in early pregnancy was 53.1 and 38.5%, respectively. The mean value of the serum 25(OH)D concentration was 19.6±7.5 ng/mL. The restricted cubic splines model showed an inverted J-shaped relationship in which the risk of GDM decreased when the 25(OH)D concentrations were ≥ 20 ng/mL. Logistic model analysis showed that 25(OH)D concentrations ≥ 30 ng/mL significantly decreased the risk of GDM (odds ratio = 0.63, 95% confidence interval: 0.45-0.89; *P* = 0.010) compared with 25(OH)D concentrations < 20 ng/ml.

**Conclusions:**

In early pregnancy, vitamin D deficiency and insufficiency were very common, and a high level of vitamin D showed protective effects against the incidence risk of GDM.

## Background

Vitamin D deficiency during pregnancy is a common public health problem [1–3]. There is increasing interest in several nonskeletal actions of vitamin D deficiency during pregnancy, including its effects on placental function, glucose homeostasis, infection and the inflammatory response [4–6]. Evidence exists for to associations of vitamin D deficiency during pregnancy with perinatal complications such as gestational diabetes mellitus (GDM), preeclampsia, spontaneous miscarriage, preterm labor, and neurodevelopmental outcomes [5, 7–12].

Evidence for a relationship between vitamin D status and GDM has been deeply investigated [13–16]. The different results in the literature can be partially attributed to differences in the cutoff of vitamin D deficiency, the timing of serum collection, the dosage of calcium supplementation, the acceptability of calcium supplementation, population dietary habits, socioeconomic status, and nutrient-associated genes.

Therefore, understanding the role of vitamin D in the development of GDM is essential for developing possible individualized supplementation suggestions to prevent GDM. The aim of the study was to assess vitamin D status in early pregnancy and understand its association with GDM risk based on data from an urban population in Shanghai, China.

## Methods

### Study population

This study was a retrospective cohort study of pregnant women conducted at Obstetrics and Gynecology Hospital, Fudan University, Shanghai, China. From June 2018 to May 2019, 9163 eligible pregnant women who underwent a 75-g oral glucose tolerance test (OGTT) during 24–28 weeks of gestation were enrolled in this study. The included participants were women who had undergone serum vitamin D measurement during their first antenatal care visit at 6 to 14 weeks of gestation. The exclusion criteria included multiple pregnancies and chronic diseases, such as type 2 diabetes mellitus, hypertension, lupus, malignancies, thyroid disorders, and acute or chronic liver diseases. Ultimately, a total of 7816 participants were included in this study. The institutional review board of the hospital approved the study protocol.

### Diagnosis of GDM

All pregnant women underwent a 75-g OGTT at 24–28 weeks of gestation. OGTTs were performed after an overnight fast for at least 8 h while the subjects were on an unrestricted diet with unlimited physical activity for at least three days. A diagnosis of GDM was made when any of the following criteria were met, according to the recommendations of the International Association of the Diabetes and Pregnancy Study Groups Consensus Panel: a fasting glucose level ≥5.1 mmol/L, 1-h glucose level ≥10.0 mmol/L, or 2-h glucose level ≥8.5 mmol/L [17].

### Data collection

Participants’ demographic information including maternal age, prepregnancy body mass index (BMI), gestational age, parity, season of blood collection, vitamin D data and other blood biomarker data at 6 to 14 weeks of gestation were extracted from the medical records in the hospital information system. The prepregnancy BMI [weight (kg)/height (m)^2^] was calculated from self-reported prepregnancy weight and height. Prepregnancy BMI categories were determined based on the recommendations of the Group of China Obesity Task Force of the Chinese Ministry of Health [18]. Gestational age was determined according to the date of the last menstrual period and was confirmed by ultrasound reports in the first trimester. The season of blood sampling at the first prenatal visit was classified as spring (from 1 March to 15 May), summer (from 16 May to 30 September), autumn (from 8 October to 30 November), and winter (from 1 December to 28 February) according to the distinct climatic features in Shanghai in 2018 and 2019.

### Blood sample collection and measurements

Fasting blood samples were collected at 6-14 weeks of pregnancy during the first antenatal care visit and at 24-28 weeks of pregnancy during the routine antenatal screening for GDM. Blood samples were immediately measured by a clinical laboratory without being frozen. Plasma glucose and triglycerides (TGs) were measured by a 7600 series automatic analyzer (Hitachi, Tokyo, Japan). Glycated haemoglobin (HbA1c) was measured using a Haemoglobin Testing System (Bio–Rad, California, USA). The serum 25(OH)D level, which is considered an accurate biomarker to indicate maternal vitamin D status [19–21], was quantified through an electrochemistry assay using a vitamin analyzer (Synovie, Chongqing, China). The lower and upper limits of 25(OH)D detection were 9.5 ng/mL and 55.5 ng/mL, respectively. The results of laboratory biomarkers were reported within 6 working hours and were quantified by the clinical laboratory of the hospital.

### Statistical analysis

Descriptive statistics in the form of frequencies (n) and percentages (%) were used to describe the maternal vitamin D status at 6–14 weeks of pregnancy, which we categorized into deficient (<20 ng/mL), insufficient (20–29.9 ng/mL) or sufficient (≥30 ng/mL), as suggested by the Endocrine Society [20]. Similar descriptive statistics were applied to describe the incidence of GDM at 24–28 weeks of pregnancy which participants were categorized into GDM group and non-GDM group [17].

Continuous data are summarized as the mean ± standard deviation (SD) or median (interquartile range) (IQR), and categorical data are displayed as percentages. The t test or ANOVA was used to test the differences in quantitative variables with a normal distribution. Nonparametric tests, including the Dunn test and Kruskal-Wallis test, were used for skewed variables. The chi-square test was used to assess differences in categorical variables. Logistic regression analyses were used to investigate the associations of serum 25(OH)D with GDM. Moreover, we used a restricted cubic spline (RCS) regression model to further analyze the dose–response relationship between vitamin D status and GDM risk. Knots were set at the 5th, 50th, and 95th percentiles and the reference value was set to the serum 25(OH)D level of 20 ng/ml.

Odds ratios (ORs), adjusted ORs (aORs) and 95% confidence intervals (CIs) were reported. Potential confounding variables (such as maternal age, prepregnancy BMI, haemoglobin levles, total cholesterol levels, lipoprotein levels) known to be associated with GDM were included in multivariable models [22, 23]. A *P* value < 0.05 was considered statistically significant in all analyses. All statistical procedures were performed using SPSS version 20.0. and R version 4.2.0.

## Results

### Distribution of serum 25(OH)D concentrations in early pregnancy

The serum 25(OH)D concentrations of pregnant women according to the categories of the relevant confounding variables are shown in Table 1. In total, the mean value of the serum 25(OH)D concentration of 7816 pregnant women, measured at 6-14 weeks of gestation, was 19.6±7.5 ng/mL. A total of 4152 (53.1%), 3011 (38.5%), and 653 (8.4%) pregnant women had deficient, insufficient, or sufficient concentrations of 25(OH)D, respectively.

**Table 1:** The serum 25(OH)D concentration of pregnant women in early pregnancy stratified by different characteristics

Characteristics	Serum 25(OH)D (ng/mL)					
	Mean	*P*	Deficiency (< 20.0)	Insufficiency (20.0-29.9)	Sufficiency (≥30.0)	*P*
Total	19.6±7.5		4152 (53.1)	3011 (38.5)	653 (8.4)	
Maternal age (Years)						
< 25	18.5±7.4	< 0.001	212 (61.3)	109 (31.5)	25 (7.2)	< 0.001
25 - 34	19.5±7.4		3305 (53.4)	2397 (38.7)	489 (7.9)	
≥35	20.3±7.7		635 (49.6)	505 (39.5)	139 (10.9)	
Parity (Number of deliveries)						
0	19.5±7.4	0.002	3218 (54.0)	2274 (38.1)	469 (7.9)	0.003
≥1	20.0±7.7		934 (50.4)	737 (39.7)	184 (9.9)	
Prepregnancy BMI (kg/m^2^)			21.4±3.0	21.3±2.8	20.9±2.6	< 0.001
< 18.5	19.8±7.7	0.004	585 (52.9)	412 (37.3)	108 (9.8)	0.002
18.5 - 23.9	19.7±7.6		2904 (52.6)	2132 (38.6)	481 (8.7)	
24.0 - 27.9	19.3±6.8		513 (54.3)	380 (40.2)	52 (5.5)	
≥28.0	18.1±6.5		150 (60.2)	87(34.9)	12 (4.8)	
Season at time of blood sampling						
Spring	22.9±7.1	< 0.001	431 (38.5)	523 (46.7)	165 (14.7)	< 0.001
Summer	22.4±6.4		1095 (37.3)	1515 (51.6)	328 (11.2)	
Autumn	18.6±7.3		814 (58.3)	487 (34.9)	96 (6.9)	
Winter	15.1±6.6		1812 (76.7)	486 (20.6)	64 (2.7)	

Abbreviations: 25(OH)D, 25-hydroxyvitamin D; BMI, body mass index. Data were presented as n (%) for categorical data, means ± SDs for parametrically distributed data.

We found a significant difference in the serum 25(OH)D concentration among pregnant women at different ages (*p* < 0.001), and the serum mean 25(OH)D concentration was obviously higher in pregnant women over 35 years of age. Multiparous women had a significantly higher serum concentration of 25(OH)D than nulliparous women (*P* =0.002). The serum 25(OH)D concentration was significantly different among women with different prepregnancy BMIs (*P* =0.004). Pregnant women who were obese before pregnancy had an obviously lower mean serum 25(OH)D concentration, while there was no significant difference in the serum 25(OH)D concentration among pregnant women who were underweight, normal weight, and overweight before pregnancy. A statistically decreasing trend of seasonal variations in the maternal 25(OH)D concentration was found (*P* < 0.001), and the mean concentration was lowest when drawn in the winter.

The prevalence of vitamin D sufficiency was higher in pregnant women over 35 years of age and multiparous pregnant women (*P* < 0.001 and *P* = 0.003). The prevalence of vitamin D deficiency was significantly higher in pregnant women who had a prepregnancy BMI ≥28 kg/m^2^ (*P* < 0.001). Vitamin D deficiency was most common in winter and least common in summer (*P* < 0.001) (Table 1).

### Participant characteristics in early pregnancy among women with and without GDM

A total of 669 (8.6%) pregnant women were diagnosed with GDM at 24-28 weeks of gestation. Table 2 shows the baseline characteristics of the participants with and without GDM in early pregnancy. Compared with the non-GDM group, the GDM group was older, had a higher prepregnancy BMI, had higher levels of TGs, HbA1c, and fasting glucose, and had a higher proportion of multiparous and overweight and obese women. Retrospectively comparing serum 25(OH)D levels in early pregnancy, the GDM group had lower mean concentrations and a lower proportion of sufficient vitamin D categories than the non-GDM group.

**Table 2:** Demographic and clinical characteristics of pregnant women with and without GDM in early pregnancy

Characteristics	All *n*=7816	GDM *n*=669	Non-GDM *n*=7147	*P*
Gestational weeks	10.7 (10.6-10.7)	10.7 (10.6-10.7)	10.7 (10.5-10.8)	0.957
Maternal age (years)	30.5±3.9	32.1±4.3	30.4±3.9	< 0.001
< 25	346 (4.4)	18 (2.7)	328 (4.6)	< 0.001
25 - 34	6191 (79.2)	466 (69.7)	5725 (80.1)	
≥35	1279 (16.4)	185 (27.7)	1094 (15.3)	
Parity (Number of deliveries)				
0	5961 (76.3)	480 (71.7)	5481 (76.7)	0.004
≥1	1855 (23.7)	189 (28.3)	1666 (23.3)	
Prepregnancy BMI	21.3±2.9	22.6±3.6	21.2±2.8	< 0.001
< 18.5	1105 (14.1)	57 (8.5)	1048 (14.7)	< 0.001
18.5 - 23.9	5517 (70.6)	419 (62.6)	5098 (71.3)	
24.0 - 27.9	945 (12.1)	138(20.6)	807(11.3)	
≥28.0	249 (3.2)	55 (8.2)	194 (2.7)	
Vitamin D concentration (ng/mL)	19.6±7.5	19.0±6.8	19.6±7.5	0.031
Deficiency (<20.0)	4152 (53.1)	361 (54.0)	3791 (53.0)	0.020
Insufficiency (20.0-29.9)	3011 (38.5)	271 (40.5)	2740 (38.3)	
Sufficiency (≥30.0)	653 (8.4)	37 (5.5)	616 (8.6)	
Triglycerides (mmol/L)	1.31±0.58	1.55±0.56	1.29±0.78	< 0.001
Glycosylated hemoglobin A1c (%)	5.10±0.27	5.26±0.29	5.08±0.26	< 0.001
Fasting blood glucose (mmol/L)	4.49±0.36	4.75±0.47	4.46±0.34	< 0.001

Data were presented as n (%) for categorical data, mean ± SD for parametrically distributed data, or median (IQRs) for nonparametrically distributed data

*Abbreviations*: *25(OH)D* 25-hydroxyvitamin D, *BMI* Body mass index, *GDM* Gestational diabetes mellitus

### Associations of maternal vitamin D status with GDM risk

**Fig. 1:**
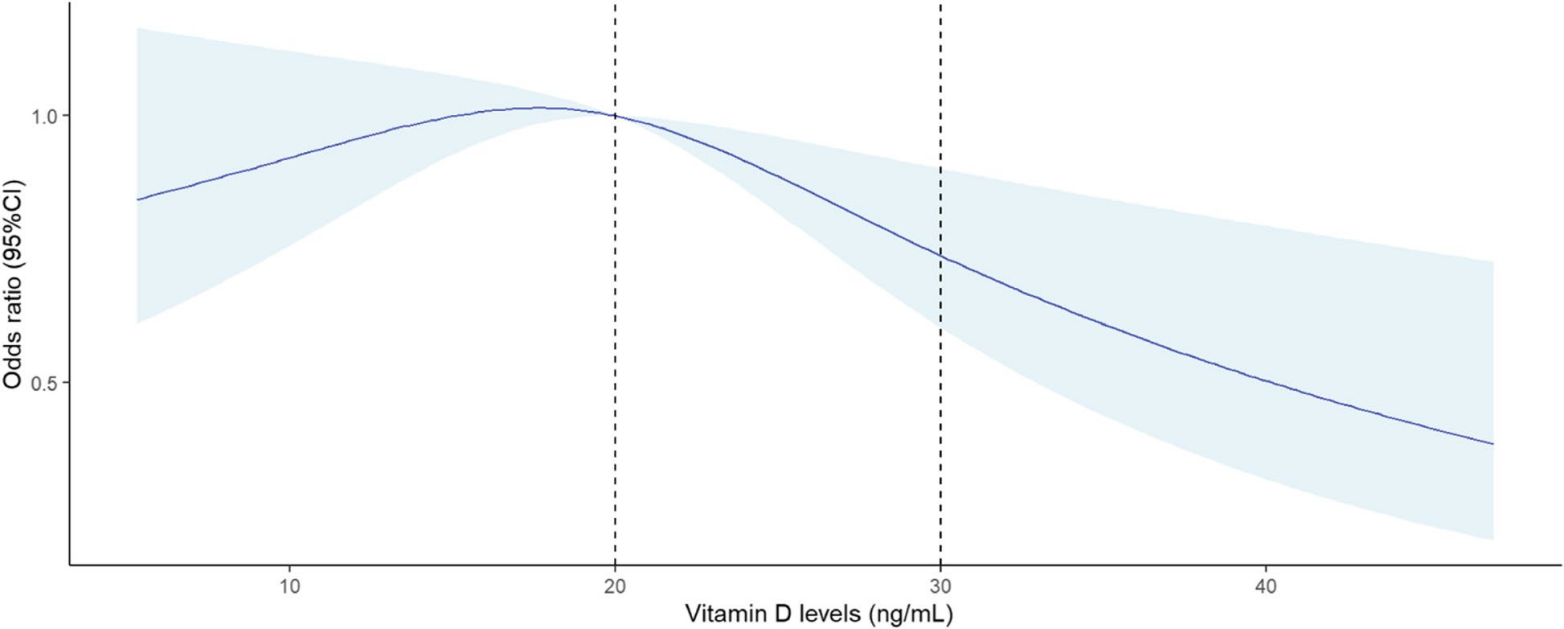
Restricted cubic spline curve association between vitamin D concertrations (ng/mL) and GDM risk, adjusted for maternal age, and prepregnancy BMI. A vitamin D level of 20 ng/mL was selected as the reference level. The blue areas represent 95% CIs. Knots were located at the 5th, 50th and 95th percentiles. Dashed vertical lines represent the category thresholds of 20 ng/mL (deficiency) and 30 ng/mL (sufficiency)

Relationship analysis was performed using 25(OH)D concentrations as continuous and categorical variables. Table 3 presents the results of the logistic regression analysis for GDM in relation to the categories of serum 25(OH)D concentrations by different models. Compared with women with 25(OH)D concentrations < 20 ng/ml, the GDM risk had no significantly difference in women with 25(OH)D concentrations ranging from 20 to 29.9 ng/ml (OR: 1.04; 95% CI: 0.88-1.23; *P* = 0.652), while the GDM risk was significantly lower in women with 25(OH)D concentrations ≥ 30 ng/ml even after adjusting for potential confounding demographic factors (aOR 0.63 [95% CI 0.45-0.91]; *P* = 0.012) or adjusting for potential confounding demographic factors and laboratory biomarkers (aOR 0.65 [95% CI 0.45-0.93]; *P* = 0.019). The RCS model suggested inverted J-shaped associations of vitamin D status with GDM after adjusting for maternal age and prepregnancy BMI (*P* for nonlinear trend = 0.012). There was no significant association between vitamin D levels and GDM when the 25(OH)D concentrations were <20 ng/mL and the risk of GDM decreased when the 25(OH)D concentrations were >20 ng/mLand continued to decrease when the 25(OH)D concentrations were >30 ng/mL (Fig. 1).

**Table 3:** Results of logistic regression analyses for development of GDM according to maternal serum vitamin D levels in early pregnancy

Serum 25(OH)D (ng/mL)	Unadjusted OR (95% CI)	*P*	Adjusted OR ^a^ (95% CI)	*P*	Adjusted OR^b^ (95% CI)	*P*
Deficiency (< 20.0)	1.00 (Reference)		1.00 (Reference)		1.00 (Reference)	
Insufficiency (20.0-29.9)	1.04 (0.88-1.23)	0.652	1.03 (0.87-1.22)	0.757	1.03 (0.86-1.23)	0.751
Sufficiency (≥ 30.0)	0.63 (0.45-0.89)	0.010	0.64 (0.45-0.91)	0.012	0.65 (0.45-0.93)	0.019

*Abbreviations*: *25(OH)D* 25-hydroxyvitamin D, *GDM* Gestational diabetes mellitus, *OR* Odds ratio, *CI* Confidence interval

^a^Adjusted for prepregnancy BMI, maternal age

^b^Adjusted for prepregnancy BMI, maternal age, glycosylated hemoglobin A1c, fasting blood glucose, triglycerides in early pregnancy

## Discussion

In this retrospective cohort study including 7816 Chinese women, vitamin D deficiency and insufficiency were common in early pregnancy. Nonlinear associations of vitamin D status with GDM were observed. A vitamin D level ≥ 20 ng/mL was a significant protective factor against incident GDM.

In our study, the mean 25(OH)D concentration was 19.6±7.5 ng/mL, 53.1% of the women had a deficient level of 25(OH)D, and only 8.4% had a sufficient concentrations of 25(OH)D according to the Endocrine Society cutoffs in early pregnancy. The rate of maternal vitamin D deficiency ranges widely. For example, the mean level of serum 25(OH)D and the prevalence of vitamin D deficiency were 30.4 ng/mL and 20.7%, respectively, in Hispanic pregnant women [14], 27.2 ng/mL and 28% in Australian and New Zealand women [24], and 13.15 ng/mL and 82.8% in Indonesian women [25]. Even in China, the rates of vitamin D deficiency also range widely in different cities. In Xi’an, the mean 25(OH)D concentration was 15.8 ng/mL in pregnant women, and 76.9% were defined as having vitamin D deficiency [26]. In the Beijing urban area in the winter, over 90% of pregnant women had vitamin D deficiency, and none had sufficient 25(OH)D concentrations [27]. In Shanghai, 69% of pregnant women had a deficient level of 25(OH)D, 22% had an insufficient level, and 9% had a sufficient level in 2012 [28]. The difference may result from many factors, including the variations in subject samples, dietary habits, determination methods, gestational weeks, assessment seasons, and longitude and latitude. Compared with Tao MF’s study in 2012 [28], vitamin D deficiency appeared to be slightly improved in Shanghai in the past decade in our study population. Micronutrient intake has improved in the Chinese population in the past decade, but natural foods that are rich in vitamin D and food and beverages that are fortified with vitamin D remain limited and greater attention and management is still needed to modify public health risks.

Little is known about factors influencing maternal vitamin D status. Major factor may be exposure to sunlight, vitamin D intake and requirements for vitamin D [15, 29, 30]. In this study, we investigated several factors. As expected, we observed that serum vitamin D levels had a seasonal variation, with the highest concentrations in the spring and summer and the lowest concentrations in the autumn and winter. We also observed that pregnant women aged ≥35 years, multiparas, and women with a lower prepregnancy BMI had higher mean serum 25(OH)D levels and a higher proportion of vitamin D sufficiency. The reason may be that vitamin D is stored in adipose tissue and accordingly reduces bioavailability [31]; therefore, women with prepregnancy overweight and boesity probably had lower 25(OH)D status. We speculate that multiparas and pregnant women of advanced age are probably more willing to supplement vitamins to obtain better pregnancy outcomes, but this speculation still needs further behavioral investigation. Similar findings were mentioned in Aji AS’s study in Indonesian women: vitamin D sufficient pregnant women were shown to be older, have a lower bodyweight before pregnancy and in the first trimester, have a lower likelihood of being nulliparous, have more outdoor activity hours and have a lower level of sunscreen application.

Our study is supportive of an increasing number of studies showing that vitamin D deficiency and insufficiency were associated with an increased risk of GDM [32, 33]. In our study, the GDM group had a lower mean level of vitamin D and lower proportion of vitamin D sufficiency in early pregnancy. Consistently, logistic regression analysis results also revealed that compared with women with 25(OH)D concentrations < 20 ng/mL, the risk was lower in women with 25(OH)D concentrations ≥30 ng/mL even after adjusting for confounding factors.

In the literature, many studies have aimed to clarify this situation. A recent study conducted in pregnant women in Hefei, China, found that GDM risk was significantly reduced only in pregnant women with 25(OH)D concentrations >20 ng/mL compared with women with 25(OH)D concentrations < 10 ng/mL [15]. Lacroix et al. found that lower first trimester 25(OH)D levels were associated with a higher risk of developing GDM even after adjustment for vitamin D confounding factors and GDM risk factors [34].

Other studies showed that the mean 25(OH)D levels and vitamin D deficiency were not significantly different between women with and without GDM [2, 35]. Similar result was also showed in our study, that the GDM risk was not significantly different in women with 25(OH)D concentrations ranging from 20 to 29.9 ng/mL compared with women with 25(OH)D concentrations < 20 ng/mL. A possible partial explanation for these conflicting data is that the effects of vitamin D status on GDM risk may be confounded and nonlinear. Our study observed inverted J-shaped associations of vitamin D levels with GDM risk, which is consistent with findings from other observational studies. Our findings, supported by Yin, W.J. et al., showed that 25(OH)D levels < 50 nmol/L (20 ng/mL) did not affect GDM risk, and a reduction in GDM risk was observed only when pregnant women had a mean 25(OH)D level that was greater than 20 ng/mL [15]. However, the threshold concentrations of 25(OH)D are different among studies. Pham, T.T.M.et al. demonstrated that GDM risk is higher at 25(OH)D levels <20 ng/mL and >35 ng/ml [2]. Salakos et al. found that GDM risk was low for women with 25(OH)D levels <10 ng/mL, increased for those with levels of 10-25 ng/mL, decreased for thosed with levels 25-40 ng/mL and increased for those with levels >40 ng/mL [36]. Recently, a systematic review and meta-analysis of prospective cohort studies found a significant U-shaped nonlinear association between serum vitamin D concentrations and the risk of developing GDM. They suggested that the risk of developing GDM is significantly high when maternal serum vitamin D concentrations are lower than 16 ng/mL or higher than 36 ng/mL. Individuals with serum vitamin D concentrations between 16 and 36 nmol/L had a significantly reduced risk of GDM [37]. The authores speculated that vitamin D levels within the cutoff range may be beneficial and that levels above or below the cutoff appear to be harmful in populations with different vitamin nutritional statuses. The strength of the association between vitamin D cutoffs and GDM risk possibly depends on the internal variations of individuals, the proportion of women with vitamin D deficiency, the prevalence of GDM, and gestational age.

Vitamin D supplementation during pregnancy could protect against GDM [3, 32]. However, there is insufficient evidence to precisely determine at what gestational age, volume, and frequency calcium supplementation should be commenced to confer this benefit. One study suggested that pregnant women taking 400-600 IU of vitamin D/d with a mean 25(OH)D concentration of 20 ng/mL had a lower risk of GDM [15]. The Endocrine Society suggests that pregnant women require at least 600 IU/d of vitamin D and recognize that at least 1500–2000 IU/d of vitamin D may be needed to maintain a blood level of 25(OH)D above 30 ng/mL [20]. In this study, vitamin D levels ≥ 20 ng/mL might be significant protective factors against GDM, and we speculate that vitamin D supplemention may be needed to maintain 25(OH)D levels above 20 ng/mL. However, it remains uncertain whether this will confer additional health benefits, and well-designed randomized controlled trials are needed to elicit the clear effect of vitamin D supplementation on the prevention of GDM.

Limitations of this study include the following: 1) This study was conducted in a single hospital with a single ethnic group; it is not possible to provide an exact, generalizable cutoff for increased GDM risk based on vitamin D levels at this stage. 2) Residual confounding cannot be ruled out, as we did not analyze data regarding vitamin D supplementation, the dietary intake of vitamin D, education levels, social data, physical activities, or family history of disease.

## Conclusions

Vitamin D deficiency and insufficiency were very common in women in Shanghai. Vitamin D levels ≥ 20 ng/mL in early pregnancy were significantly association with a lower risk of GDM. Individualized vitamin D supplementation before or during pregnancy should be considered to minimize the risk of GDM.

## Data Availability

The datasets used and/or analyzed during the current study are available from the corresponding author upon reasonable request.

## Abbreviations

GDM: Gestational diabetes mellitus
25(OH)D: 25-hydroxy vitamin D
BMI: Body mass index
CI: Confidence interval
OR: Odds ratio
